# Factors Affecting Visual Outcomes Following Scleral-Fixated Intraocular Lens Implantation at a Tertiary Care Center in Eastern India

**DOI:** 10.7759/cureus.76154

**Published:** 2024-12-21

**Authors:** Neha Kumari, Abhishek Anand, Rajnee Sinha, Mobashir Ali, Shivani Sinha, Bibhuti Sinha

**Affiliations:** 1 Ophthalmology, Indira Gandhi Institute of Medical Sciences, Patna, IND

**Keywords:** aphakia, scleral-fixated intraocular lens, subluxated lens, sutureless, trauma

## Abstract

Background: Lens implantation becomes a major concern in patients lacking posterior capsular support, but various methods are available for rehabilitation. In such patients, scleral-fixated intraocular lens (SFIOL) implantation is preferred due to its fewer complications and better simulation of the natural lens position. In this non-randomized retrospective clinical study, we aimed to assess visual outcomes after sutureless SFIOL implantation in aphakic patients and factors affecting visual outcomes.

Materials and methods: The study was conducted at the Indira Gandhi Institute of Medical Sciences, Patna, Bihar, India, wherein a retrospective data analysis of hospital medical records of aphakic cases who underwent SFIOL implantation from January 2020 to December 2022 was performed. Patients with poor capsular and iris support, lens subluxation of >180°, dislocated natural crystalline lens, or intraocular lens were included in the study. Preoperative data such as aphakia etiology, pre-op best-corrected visual acuity (BCVA), intraocular pressure (IOP), and any other ocular pathology, as well as postoperative data (i.e., BCVA, IOP, haptic status, and complications), were collected and analyzed.

Results: A total of 45 patients underwent non-sutured multipiece SFIOL implantation. The average preoperative BCVA was 1.73±0.24 (in LogMAR, logarithm of the minimum angle of resolution), which improved to 0.87±0.27 six months after surgery. Out of 45 patients, 41 (91.1%) reported increased BCVA. The most common intraoperative and postoperative complications were astigmatism, raised IOP, and corneal edema. Four (8.9%) patients had no change in BCVA due to pre-existing conditions. No patient required a second surgery for retinal detachment or dislocation of SFIOL. Two (4.4%) patients underwent laser treatment for a retinal break found incidentally during surgery.

Conclusion: Sutureless SFIOL demonstrates predictable outcomes post-aphakia. Patient outcomes ultimately depend on the presence of postoperative astigmatism and coexistent anterior and posterior segment pathologies. Surgeons should take appropriate care to avoid IOL tilt and postoperative astigmatism.

## Introduction

Aphakia is defined as the absence of a lens in the pupillary area. The most common cause of aphakia is complicated cataract surgery, followed by ocular trauma and congenital aphakia [1]. Other conditions with similar management protocols to that of aphakia include ectopia lentis, which is acquired, and familial displacement of the crystalline lens from the patellar fossa [2]. Although ectopia lentis and aphakia are completely different entities, they are managed similarly.

Multiple conservative and surgical options are available for managing aphakia. Conservative approaches include aphakic spectacles and contact lenses, whereas surgical methods include anterior chamber (AC) intraocular lenses (IOLs), iris-claw IOLs, and scleral-fixated intraocular lenses (SFIOLs) [1].

Aphakic spectacles are easy to prescribe but have multiple inherent disadvantages, such as poor optical quality, image magnification, a constricted visual field, a jack-in-the-box phenomenon, and increased nystagmus amplitude. Contact lenses can also be used and are generally preferred in infants whenever an IOL cannot be placed, but they require meticulous care.

Three types of contact lenses are mainly used to correct aphakia: rigid gas-permeable, silicone elastomer, and hydrogel lenses. Although rigid gas-permeable lenses have the best performance, they are known for poor compliance during long-term use and can cause serious adverse events, such as ocular irritation and infection [3].

All of the abovementioned complications of contact lenses can be avoided with surgical corrections of aphakia, which are physiological in nature and exhibit high patient compliance. Current surgical methods to correct aphakia in the absence of capsular support include AC IOL, anteriorly or posteriorly iris-fixated IOL, and SFIOL [4].

To the best of our knowledge, no study has evaluated the long-term visual prognosis and complications of SFIOL implantation. Furthermore, results can vary dramatically, depending on the surgical technique employed and the aphakia etiology. Therefore, this study aimed to assess visual outcomes after sutureless SFIOL implantation in aphakic patients and factors affecting visual outcomes.

## Materials and methods

This non-randomized retrospective clinical study was conducted at the Indira Gandhi Institute of Medical Sciences in Patna, Bihar, India. The study was approved by the institutional ethics committee, and it was performed in full compliance with the tenets of the Declaration of Helsinki.

Medical records of patients who underwent SFIOL surgery by a single surgeon from January 2020 to December 2022 were scrutinized. Data of patients fulfilling the inclusion and exclusion criteria were collected with a predesigned proforma.

All aphakic patients without capsular support, as well as patients with subluxated lenses (>180°), dislocated natural crystalline lenses, or IOL between the ages of five and 80 years of any sex, were included in the study. Patients with moderate to severe dry eye or having any ocular pathologic condition that impairs visual function (e.g., any suspected corneal dystrophies or abnormalities, strabismus, hazy cornea, glaucoma, diabetes mellitus, any retinal pathology, or optic nerve atrophy) were excluded from the study.

Parameters noted included pre-treatment findings (aphakia etiology, best-corrected visual acuity (BCVA), keratometry readings, applanation tonometry readings, and other ocular pathologies), treatment findings (procedure done with any intraoperative complications, time elapsed since surgery), and post-treatment clinical course (residual refractive error, pigmentation over the lens, haptic status, centration of lens, keratometry reading, applanation tonometry readings, and immediate, short-, and late-term complications).

Standard surgical procedure

All procedures were performed by a single operating surgeon. In patients requiring concurrent pars plana vitrectomy with SFIOL surgery, taking aseptic and antiseptic precautions, >180° peritomy was done. Using a marker pen, a toric marker was used to mark diametrically opposite ends on the limbus. Two square scleral pockets were dissected using a crescent at diametrically opposite ends. An AC maintainer was used in most cases rather than using an infusion at the pars plana, as we have found that the former provides more stability and induces less prolapse of the iris when we introduce the lens or perform any manipulation in the AC. After establishing the AC maintainer, just adjacent to the scleral groove, 1.5 mm behind the limbus, a stab incision was given using a 23-gauge microvitroretinal blade. Two paracenteses were created at the 10 o'clock and 2 o'clock positions of the limbus. After completing the pars plana vitrectomy, a foldable Indian-made multipiece IOL was placed either through the limbal or sclerocorneal groove. At the point of introduction, the leading haptic was grasped at the tip by serrated straight forceps or SFIOL forceps.

In addition, as we pushed the lens through the cartridge, the leading haptic was also simultaneously pulled so that the haptic glided out and extruded through the intended site. McPherson forceps were used to grasp the trailing haptic, which was tucked at the corneal incision to prevent displacement. In cases where the trailing haptic came into the AC, the former was externalized using the handshake technique. Each haptic was externalized and fixed. The main port was closed with a 10-0 nylon suture, and the peritomy was closed. Postoperatively, all patients received antibiotics, corticosteroids, cycloplegic, and lubricating eye drops. Anti-glaucoma medication was given to patients with increased IOP.

Data analysis

Data were analyzed using IBM SPSS Statistics for Windows, Version 20 (Released 2011; IBM Corp., Armonk, New York). Descriptive statistics were applied for demographic variables. Student's 𝑡-test was used to assess differences in BCVA between groups, where statistical significance was defined as p<0.05.

## Results

A total of 45 patients who underwent sutureless SFIOL implantation during the study period were included in this study. Table 1 summarizes the demographic details of these patients. The average age of the patients was 43.72±21.91 years, with the youngest being seven years old. The study sample was predominantly male, comprising 29 (64.4%) male and 16 (35.5%) female patients. The mean follow-up period was 11.5±4.2 months. The most common underlying cause for SFIOL implantation was trauma, accounting for 25 (55.6%) patients, of which 14 (31.1%) had closed-globe injury, and the remaining 11 (24.4%) had open-globe injury. Four (8.9%) patients had clear corneal tears, six (13.33%) had corneoscleral tears, and one (2.2%) had pure scleral tears.

**Table 1:** Demographic characteristics of the study participants (N=45) SD: standard deviation

Demographic Variable	Category	Value
Age	Mean±SD	43.72±21.91 years
	Range	7–74 years
Sex	Male	29 (64.4%)
	Female	16 (35.5%)
Eye involved	Right	30 (66.7%)
	Left	15 (33.3%)
Follow-up	Mean±SD	11.5±4.2 months

A total of 15 (33.33%) patients underwent SFIOL implantation from iatrogenic causes as a complication from previous surgeries, of which six (13.3%) patients had a huge posterior capsule rent, three (6.7%) had nucleus drop, and four (8.9%) had IOL drop (Table 2). The mean preoperative BCVA of the patients was 1.73±0.24 (in LogMAR, logarithm of the minimum angle of resolution). Postoperatively, the mean BCVA was 0.98±0.25, 0.83±0.26, and 0.87±0.27 at one week, one month, and six months, respectively (Table 3).

**Table 2:** Underlying etiology of cases (N=45) IOL: intraocular lens

Etiology	n (%)
Aphakia	14 (31.11%)
1a. Iatrogenic	6 (13.3%)
1b. Traumatic	8 (17.8%)
Posteriorly dislocated crystalline lens	8 (17.8%)
2a. Traumatic	7 (15.6%)
2b. Spontaneous	1 (2.2%)
Anteriorly dislocated lens	1 (2.2%)
Subluxated cataractous lens	6 (13.33%)
4a. Trauma	5 (11.1%)
4b. Spontaneous	1 (2.2%)
Subluxated IOL	1 (2.2%)
Dislocated IOL	4 (8.9%)
Dropped nucleus	3 (6.7%)
Opacified IOL	1 (2.2%)
Traumatic cataract with inadequate capsular support	7 (15.6%)

**Table 3:** Mean BCVA values of the study participants (N=45) *p-values determined using the Student's t-test BCVA: best-corrected visual acuity; LogMAR: logarithm of the minimum angle of resolution

Period	BCVA (in LogMAR)	p-value*
Preoperative	1.73±0.24	-
Postoperative day 1	1.07±0.26	<0.01
Postoperative day 7	0.98±0.25	<0.01
Postoperative day 28	0.83±0.26	<0.01
Postoperative month 6	0.87±0.27	<0.01

The most common intraoperative IOL-related complication observed during surgery was the breaking of the IOL at the haptic optic junction and hyphema (n=3, 6.7%). In the immediate postoperative period, the most common complications were raised IOP (n=12, 26.7%) and corneal edema (n=11, 24.4%), followed by vitreous hemorrhage (n=8, 17.8%). None of the patients required surgical intervention for vitreous hemorrhage. Raised IOP was managed by IOP-lowering agents. However, IOP could not be stabilized long-term in five (11.1%) patients who required continuous use of anti-glaucoma medications.

In the early postoperative period, the most common complication was pupil-shape abnormalities (n=8, 17.8%), followed by IOL decentration (n=4, 8.9%). In the late postoperative period, the most common complication was astigmatism (n=17, 37.8%), followed by pigment dispersion (n=8, 17.8%). Corneal decompensation was noted in three (6.7%) patients who were managed with a deturgescent agent and registered for keratoplasty. Intraoperatively, a retinal break was noted incidentally in two (4.4%) patients, which was sealed with a laser (Table 4).

**Table 4:** Intraoperative and postoperative complications (N=45) IOL: intraocular lens

Period	Complication	n (%)
Intraoperative	IOL-related	3 (6.7%)
	Hyphema	3 (6.7%)
	Retinal break	2 (4.4%)
Immediate postoperative	IOL-related	3 (6.7%)
	Corneal edema	11 (24.4%)
	Hyphema	4 (8.9%)
	Raised intraocular pressure	12 (26.7 %)
	Vitreous hemorrhage	8 (17.8%)
Early postoperative	IOL decentration	4 (8.9%)
	Pupil abnormalities	8 (17.8%)
	Macular edema	0 (0%)
Late postoperative	Endophthalmitis	0 (0%)
	Haptic related	4 (8.9%)
	Corneal decompensation	3 (6.7%)
	Glaucoma	5 (11.1%)
	Pigment dispersion	8 (17.8%)
	Astigmatism	17 (37.8%)
	Retinal detachment	1 (2.2%)

None of the patients developed hypotony, choroidal detachment, cystoid macular edema, or endophthalmitis. In addition, none of the cases required re-surgery for retinal detachment, dislocation of SFIOL, or other complications. However, two (4.4%) patients had to undergo glaucoma filtration surgery because of uncontrolled IOP despite being on maximal medications.

## Discussion

The management of aphakia becomes quite challenging in the absence of zonular or capsular support, with only a few options for treatment, such as anterior chamber IOL, iris-fixated IOL, and SFIOL. Among these options, SFIOL simulates the natural position of the lens. Although SFIOL requires a longer surgical time and surgical expertise, once learned, the procedure can provide patients with better vision while minimizing complications.

The surgical approach depends on the surgeons' expertise in particular techniques and coexisting ocular pathology, such as corneal health, as well as anterior segment, pupillary, and iris morphology and integrity, among others. Overall, all techniques have predictable and good long-term visual outcomes in expert hands. Anterior chamber IOL and iris-fixated IOL appear to have a shorter learning curve; however, they do have a few issues, such as increased endothelial cell loss, corneal decompensation, and the incidence of recurrent uveitis. Iris-claw lenses have longer visual rehabilitation and can cause ovalization of the pupil and generalized or localized iris atrophy. SFIOL has a longer learning curve but tends to be more physiologically placed than other IOLs. Complications of sutureless SFIOL include haptic extrusion, lens tilt, complex astigmatism, postoperative hypotony, and vitreous hemorrhage. The main advantage of SFIOL over other IOLs is that it can be placed in compromised corneas, as well as with coexisting iris trauma or iridodialysis.

Surgical outcomes also depend on the etiology of aphakia. In cases of trauma, surgery becomes even more challenging, as the surgeon must overcome complications such as media haze and retinal breaks, which are relatively more common in cases of traumatic aphakia than other etiologies.

SFIOL is broadly classified as either sutured or sutureless. The fixation point of the haptic in sutured SFIOL can be two-point to four-point fixation. In two-point fixation, there is more risk of IOL tilt and decenteration. The limitations of sutured SFIOL include surgical expertise, prolonged surgical time, suture-induced inflammation, suture degradation, and delayed IOL subluxation or dislocation due to broken sutures [5]. These complications are comparatively less in sutureless SFIOL, making it a more preferred technique for SFIOL implantation [6]. Techniques are categorized into two groups based on a scleral tunnel with or without a scleral flap.

The history of sutured SFIOL can be traced back to Malbran et al., who first published literature on the management of aphakia by sutured SFIOL [7]. The ab externo concept was then popularized by Lewis in 1991, who developed the technique to bury the suture knots with the help of scleral flaps [8]. To bury sutures, various methods, such as the Z-suture, have been utilized by surgeons. Common complications of sutured SFIOL include suture breakage and lens dislocation, and rare complications include endophthalmitis, vitreous hemorrhage, retinal detachment, and suprachoroidal hemorrhage.

Sutureless SFIOL was first done and reported by Scharioth et al. [9]. Subsequently, various modifications were introduced by Prenner et al. [10] and Abbey et al. [11]. Recently, Agarwal et al. introduced a popular modification of sutureless SFIOL, wherein fibrin glue was used to seal the scleral bed and secure haptics. The sutureless technique avoids the complications caused by suture placement [12].

Compared to other studies, BCVA was found to be poor both preoperatively and postoperatively in the current study. This difference could be due to primarily treating more traumatic cases [1,4].

The main postoperative complications reported in various SFIOL studies include astigmatism, retinal detachment, corneal edema, glaucoma, and vitreous hemorrhage [13-16]. The most common intraoperative complications are haptic rebound and IOL drop, which were not found in our study. The AC maintainer, used as an infusion system, not only helped to maintain AC but also reduced iris prolapse found in the pars plana infusion system.

In our study, an immediate rise in IOP was found in 12 (26.7%) patients, and five (11.1%) patients developed glaucoma as a long-term complication. Despite maximal medical therapy, IOP was uncontrolled in two (4.4%) patients, who were further treated with glaucoma filtration surgery.

Out of 45 patients, in the immediate postoperative period, corneal edema was noted in 11 (24.4%) patients, which can be mainly attributed to damage to endothelial cells and raised IOP. The corneal edema resolved in eight (17.8%) patients but persisted in three (6.7%) patients. Long-term, chronic edema can eventually cause scarring of the cornea, resulting in severe vision loss.

In our study, the most common cause of visual impairment after surgery was astigmatism (n=17, 37.8%), resulting from tight sutures, corneal scars due to trauma, and lens decentration. Epiretinal membrane formation, which often requires a longer follow-up period, was not seen in any case.

In our study, no incidence of hypotony, choroidal detachment, cystoid macular edema, or endophthalmitis was noted, as observed in many other studies [16-18]. However, some authors have reported these complications, such as Deb et al., who found a slightly elevated rate of cystoid macular edema (13.33%) because of taking traumatic cases [19].

Study limitations

This was a retrospective study with a small sample size. A prospective study would have been more useful in collecting more data. In addition, specular biomicroscopy for endothelial cell count was not performed. As the outcome of surgery also depends on the etiology of aphakia, future studies could incorporate etiological analysis, which was not performed because of the paucity of data. Finally, the prognosis also depends on the age group of the patients; however, as we treat a few pediatric patients, a separate age-wise analysis was not performed.

## Conclusions

This study highlights the various intraoperative and postoperative complications that can occur in sutureless SFIOL implantation. The most common causes of long-term poor vision were posterior pathology and secondary glaucoma, leading to optic nerve damage. Further studies are needed to reduce both short- and long-term complications and enhance visual outcomes.
